# Resveratrol Reduces Lipid Accumulation through Upregulating the Expression of MicroRNAs Regulating Fatty Acid Bet Oxidation in Liver Cells: Evidence from *In-vivo* and *In-vitro* Studies

**DOI:** 10.22037/ijpr.2019.111745.13332

**Published:** 2020

**Authors:** Mehdi Koushki, Mina Zare, Maryam Shabani, Maryam Teimouri, Hossein Hosseini, Reyhaneh Babaei Khorzoughi, Reza Meshkani

**Affiliations:** a *Department of Clinical Biochemistry, Faculty of Medicine, Tehran University of Medical Sciences, Tehran, Iran. *; b *Recombinant Protein Laboratory, Department of Biochemistry, Shiraz University of Medical Sciences, Shiraz, Iran.*

**Keywords:** miR107, miR10b, Resveratrol, Lipogenesis, Liver, Fatty acid beta oxidation

## Abstract

MicroRNAs have been shown to regulate lipogenesis in liver. The aim of the present study was to investigate whether the effects of resveratrol (RSV) on lipogenesis are associated with the changes in the expression of two miRNAs (miR-107 and miR-10b) that regulate lipogenic pathways. 30 wild type C57BL/6j male mice were randomly fed three diets: a standard chow diet (ND), a high fat diet (HFD, 60% fat) and the high fat diet supplemented with 0.4% RSV (HFD-RSV) for 16 weeks. HepG2 cells were treated with high glucose (33 mM) and RSV (20 µM) for 24 h. The expression of the genes and miRNAs were measured by real-time PCR. Triglyceride level was increased in the liver of mice and HepG2 cells. In both animal and *In-vitro* experiments, triglyceride level was significantly decreased in groups treated with RSV. The expression of the miR-107 and miR-10b was significantly upregulated in the liver of HFD mice, whereas HFD-RSV group demonstrated a significant lower expression of both miRNAs compared to HFD group. In addition, RSV treatment significantly upregulated the expression of CPT-1a and PPARα genes in the liver of HFD mice. Moreover, treatment with RSV could reduce the expression of miR-107 and miR-10b and increase the expression of CPT-1a and PPARα in HG-treated HepG2 cells. These evidence, as a whole, suggest that RSV could exert its anti-lipogenic effect partially through alterations in the expression of miR-107 and miR-10b in liver cells.

## Introduction

Obesity is associated with low-grade systemic inflammation, insulin resistance and hyperlipidemia. Obesity increase the risk of chronic diseases such as non-alcoholic fatty liver disease (NAFLD), type 2 diabetes and cardiovascular disease (CVD) (1-3). NAFLD is characterized by the excessive accumulation of fat in the liver, which typically progresses to non-alcoholic steatohepatitis (NASH), fibrosis, cirrhosis and finally liver failure (4). The prevalence of NAFLD is an increasing clinical problem worldwide, which is estimated to be between 14 to 24% (5, 6).

MicroRNAs (miRNAs) are small non-coding RNA with 19-25 nucleotides, which regulate the gene expression by binding to the 3^´^-untranslated regions (3-UTR) of target mRNA (7, 8). miRNAs regulate several features of cellular activity, from differentiation to proliferation and apoptosis (9). Recently, it was reported that miRNAs had a considerable effect on lipid metabolism and alterations in the levels of these small molecules might be involved in the pathogenesis NAFLD. In this regard, miR-107 significantly decreased the levels of the mitochondrial β-oxidation enzymes leading to promotion of hepatic lipid accumulation and impairing glucose tolerance *In-vivo* (10). In addition, it was suggested that CPT-1a protein expression was down-regulated by the over-expression of miRNA-107 in AML-12 hepatocytes (11) . It was also demonstrated that miRNA-10b regulates cellular steatosis level by targeting peroxisome proliferator-activated receptor α (PPAR-α) expression. miR10b was upregulated in steatotic L02 cells and PPAR-α was the direct target of miRNA-10b as it showed significantly changed protein expression in steatotic L02 cells transfected with pre-miRNA-10b and anti-miRNA-10b (12) .

Resveratrol (3,4,5 trihydroxystilbene), (RSV) a natural polyphenol, is mainly found in grapes, red wine and berries(13-15). In the last few years, RSV effects on prevention of chronic conditions such as neurodegenerative disorders, CVDs, cancer, diabetes, and metabolic diseases have been demonstrated (16) . The evidence has demonstrated that the beneficial effect of RSV on health is mediated through its antioxidant and anti-inflammatory properties, cardioprotective, and neuroprotective activities (17). In addition, it was reported that RSV affected lipid metabolism. In this regard, the evidence demonstrates that the beneficial effect of RSV on liver steatosis is mediated through decreasing adipogenesis (18) and lipogenesis (19) , increasing lipolysis (20), and enhancing mitochondrial fatty acid β-oxidation(21, 22). However, little is known concerning the potential involvement of miRNAs on changes induced by RSV in the lipogenic pathways. In this context, the aim of the present study was to determine whether, the reduction in liver fat by RSV was correlated with the changes in the expression of miRNA-10b and miRNA-107, as well as their target genes *In-vitro* and *In-vivo* models of lipid steatosis.

## Experimental

Male C57BL/6J mice weighing 10-20g were purchased from Pasteur Institute of Iran (Tehran, Iran). The HepG2 cell line was purchased from Iranian Biological Resource Center (Tehran, Iran). D-glucose, D-mannitol, RSV, Oil red O stain were obtained from Sigma chemicals (St, Louis, MO, USA). miRNeasy mini, miScript II PCR (Qiagen, Biorain, Iran) and miScript SYBR Green PCR kits were purchased from Qiagen. (Qiagen, Biorain, Iran). Hybrid R Blood RNA purification kit was purchased from GeneAll Biotechnology CO. RevertAid first strand cDNA synthesis from ThermoFisher Scientific.

*Animal experiments*

This study was carried out equally with the guidelines of the institutional animal care of Tehran University of Medical Sciences. The protocol was approved by Animal Ethics Committee. Male C57BL/6J mice weighing 10-20 g, 6-8 week-old were kept under standard laboratory conditions 22 ± 0.5 ^°^C, 40-70% humidity and 12h/12h light/dark cycle, with food and water ad libitum. After a one-week adaptation period, mice were randomly divided into three groups (10 in each group), and fed the following experimental diets for 16 weeks, standard chow diet group (ND, 10 kcal% fat), high fat diet (60% high-fat diet, Research Diet, New Brunswick, NJ, USA, Country, D12492) and HFD-supplemented with 0.4% RSV (HFD-RSV) group. During this treatment period, the body weight was recorded weekly. At the end of 16 weeks, Animals were sacrificed under anesthesia by intraperitoneally administering ketamine and xylazine following a 12-h fasting period. Lastly, liver samples were removed from three different groups, immediately frozen in liquid nitrogen and then stored at -70 ^°^C until use. 

*Cell culture*

HepG2 cells were cultured in Dulbecco ʼs Modified Eagle ʼs Medium (DMEM) supplemented with 10% FBS (fetal bovine serum), 100 U/mL penicillin and 100 g/mL streptomycin at 37 °C in a humidified atmosphere containing 5% CO_2_. All experiments were performed when the cells reached about 50-70% confluence. HepG2 cells were treated with high glucose (33 mM) to induce steatosis and a dose of 20 µM of RSV for 24 h. 

*Oil Red O staining*

Hepatic lipid accumulation was stained using Oil red O method as described previously (23). After treatment, the cells were washed three times with PBS and then they were incubated with formalin (10% formaldehyde, 90% PBS) for 15 min. After fixing, cells were washed 3-4 times with distilled water. Oil red O solution (2 mL) was then added to each well and incubated at 37 °C incubator for 15 min. The cells were then washed several times with ddH_2_O for removing background color until the solution became clear. After being dehydrated, the cells were evaluated under a light microscope. Isopropanol (200 µL) was added to each well and after shaking and incubating at room temperature for 15 min, the extract was collected and transferred to a 96-well plate to measure the absorbance at 510 nm. 

*Measurement of triglyceride level*

Briefly, the cells or liver tissues were washed three times with PBS. RIPA working solution was added to the cells and tissues and incubated for 10-20 min on ice. Then, HepG2 cells and liver tissues were homogenized by oltrasonication methods. After centrifugation, a mixture of chloroform and methanol (2:1) was added to the supernatants. After mixing and then incubating for 30 min, samples were centrifuged at 12000g, for 5 min. The upper solution was removed and the lower solution was dried at 70 °C. Then, after adding PBS to dried solution, triglyceride content was determined using commercial kit (Pars Azmon, Iran). The results were normalized against total protein level. Total protein level was measured by BCA (bicinconic acid) kit.

*RNA extraction and Real-time quantitative PCR*

Total RNA from liver tissue and HepG2 cells were isolated using GeneAll RibospinTM kit (GeneAll Biotechnology, South Korea). miRNA was isolated with miRNeasy mini kit (Qiagen, Biorain, Iran). The quality and quantity of the RNA and miRNA were assessed by electrophoresis and Nanodrop 2000. Complementary DNA (cDNA) was reverse transcribed using a RevertAid First Strand cDNA Synthesis Kit (Thermo Fisher Scientific). The expression of miR-107 and miR-10b and CPT-1a and PPARα genes were measured by quantitative Real-time PCR using miScript SYBR Green PCR kit and SYBR Green master mix. The normalization was carried out using U6 small nuclear RNA (RNU6) for miRNAs and ß-actin for the genes, respectively. The sequences of the primers used in the study are in Table 1. The ΔΔCt method was used to compare the expression of miRNAs and genes between the groups.

*Statistical analysis*

Data are presented as mean ± SD. Statistical analysis was performed using SPSS 21.0 (SPSS Inc. Chicago, IL, USA). Results were analyzed by ANOVA. Differences among the groups were attained by Tukey multiple comparisons. If the p value was less than 0.05, the difference was considered statistically significant. 

## Result

*In-vivo study*

**Figure 1:**
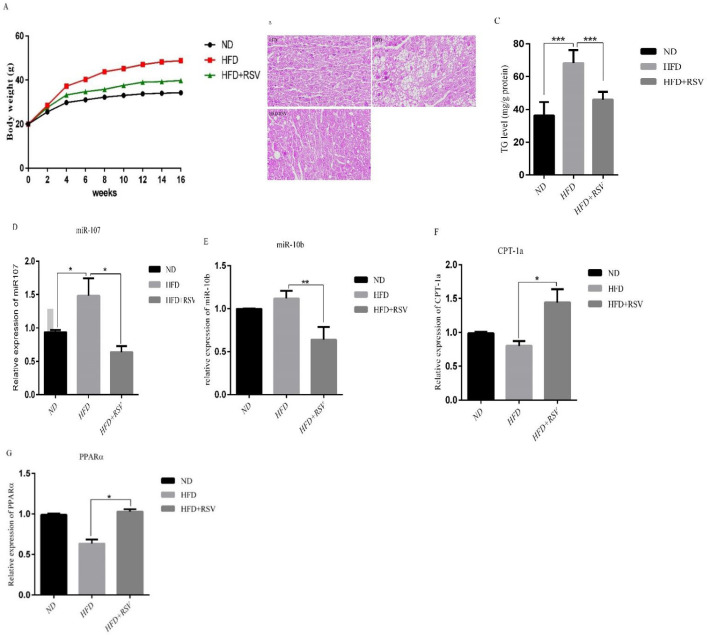
Effects of HFD and HFD supplemented with 0.4% resveratrol (RSV) on A) Body weight, B) Representative H & E staining from paraffin-embedded liver tissues in ND, HFD and HFD-RSV mice. HFD induced a drastic liver steatosis that revealed with increasing of lipid droplets, C) TG levels, the expression levels of D) miR-107, E) miR-10b and their- related target genes F) CPT-1a and G) PPARα in liver tissue. Data presented as means ± SD. Each experiment was repeated three times. Bars represent standard deviation. (*P< 0.05, **P<0.01). ND, Normal diet; HFD, High Fat Diet; HFD-RSV, High Fat Diet-Resveratrol

To investigate the effects of resveratrol on miRNAs in the liver, we treated mice with NFD, HFD and HFD+RSV diets for 16 weeks. Body weight gain was significantly reduced in liver of mice treated with REV compared with HFD group alone (Figure 1A). The histological examinations of liver revealed lipid droplet accumulation in HFD group compared with HFD-RSV and control groups (Figure 1B). To confirm the effect of RSV on lipid accumulation, we measured the triglyceride (TG) levels. The levels of TG in HFD group was significantly higher than that of the NFD group and RSV treatment could significantly decrease TG levels in HFD mice (Figure 1C). We also found that the expression of two miRNAs analyzed miR-107 and miR-10b, were significantly upregulated in the liver of mice treated with HFD (Figures 1D, 1E). Importantly, HFD-RSV group demonstrated a significant lower expression of both miRNAs compared to HFD group. A reduced expression of the target genes for miR-107 and miR-10b, CPT-1a and PPARα, respectively, was observed in the liver of HFD group compared to HFD mice, whereas, RSV treatment significantly upregulated the expression of CPT-1a and PPARα genes in the liver of HFD mice (Figures 1F-G). 

*In* *-* *vitro study*

**Figure. 2:**
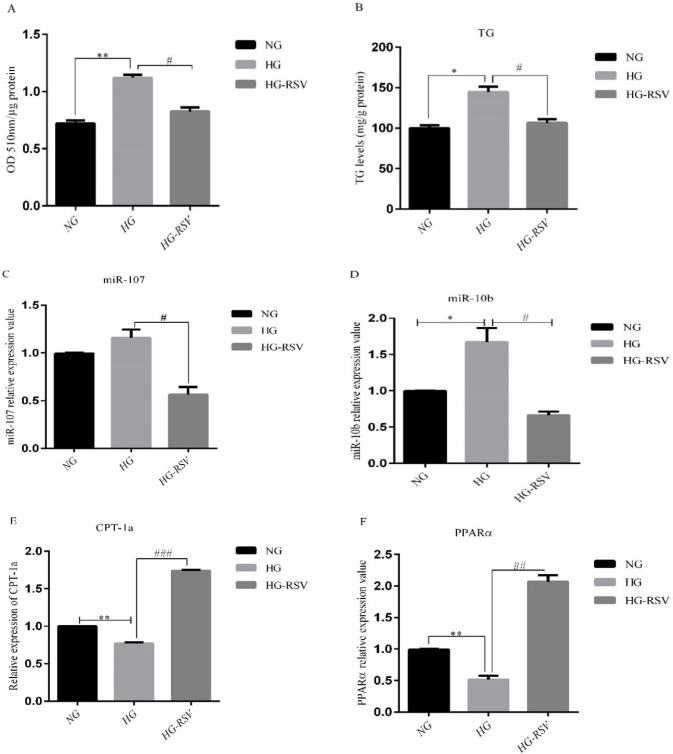
Effects of resveratrol (20 µM) on A) ORO-based quantitative assay, B) TG levels and the expression levels of C) miR-107, D) miR-10b and their target genes, E) CPT-1a and F) PPARα in HepG2 cells treated with high glucose. Values are means ± SD (Standard Deviation) of three independent experiments. The asterisks represent differences versus NG and HG (*P < 0.05, **P < 0.01) and HG compared with HG-RSV (^#^*P *<0.05, ^##^*P *< 0.01, ^###^*P *<0.001). NG, Normal Glucose; HG, High Glucose; HG-RSV, High Glucose Resveratrol

To confirm our finding from *In-vivo* experiments, we treated HepG2 cells with glucose 33mM and RSV 20 µM for 24 h. The doses of the treatments were selected based on the previous studies (24, 25). The alterations of cellular lipid accumulation were evaluated using Oil Red O (ORO) staining. The results showed that intracellular lipids were significantly increased in high glucose (HG)-treated HepG2 cells, whereas, intracellular lipid content was significantly reduced in HG group following RSV treatment (Figure 2A). It was also found that RSV treatment could significantly reduce HG- induced TG levels in HepG2 cells (Figure 2B). To gain further insight into the mechanisms of the reducing effect of resveratrol on lipid accumulation, we measured the expression of miR-10b and miR-107 in HepG2 cells. While HG treatment significantly upregulated the expression of miR-10b and miR-107, RSV treatment reduced the expression of these miRNAs in HG-treated cells (Figures 2C-D). We also found that CPT-1a and PPARα mRNA expression levels significantly decreased in the HG group, whereas, treatment with RSV resulted in a marked increase in the levels of these genes in HG-treated HepG2 cells (Figures 2E-F).

## Discussion

Fat accumulation in the liver increases the risks of NAFLD and NASH (26). Importantly, hepatic steatosis is always conjugated with other disease such as diabetes (27). Previous published studies have demonstrated an increased lipogenesis and a decreased fatty acid β-oxidation in hepatic steatosis and these processes are tightly controlled by a number of genes including CPT-1a and PARs such as PPARα. These genes have been shown to be regulated by miRNAs such as miR-107 and miR-10b. Studies have reported specific miRNA signatures during the progression of NAFLD using a HFD animal models (28) and therefore modulation of miRNA expression could be a potential therapeutic target for treatment of this disease. In the present study, we aimed to investigate whether the anti-lipogenic effects of RSV is associated with changes in the expression of miRNAs regulating lipid accumulation including miR-107 and miR-10b and their target genes in the liver cells.

*We first* successfully developed *In-vivo* and *In-vitro* models of NAFLD in mice and HepG2 cells, respectively. Our results demonstrated a greater lipid accumulation in liver tissue of the HFD mice compared to control group. Resveratrol supplementation in HFD treated mice was effective in decreasing lipid accumulation and TG level in hepatocytes. In addition, our data from histopathological examination of the livers showed a significant increase in the number and size of fatty hepatocytes upon HFD administration and RSV supplementation could returned these changes to normal levels. For validation of results in animal study, we conducted *In-vitro* model using HepG2 cells treated with high concentration of glucose (33 mM). In HepG2 cells model, we observed significantly decreased levels of miR-107 and miR-10b and significant increased levels of CPT1-1a and PPARα gene expressions in HepG2 cells treated with HG-RSV compared with HG treated group. The results of ORO staining and TG level examination showed markedly accumulated intracellular lipids and higher TG levels in HG-treated HepG2 cell model. 

miR-107 is an intronic miRNA, and exists within the intron of the pantothenate kinase (PANK) gene. This miRNA regulates several cellular processes such as development, oncogenesis, hypoxia, platelet reactivity metabolism and angiogenesis (10). Importantly, a role of miR-107 in acetyl-CoA and lipid metabolism have also suggested (29). In this regard, several studies have consistently reported elevated miR-107 levels in the livers of different models of NAFLD (30-33). At the molecular level, it was reported that miR-107 promoted hepatic lipid accumulation by suppressing mitochondrial β-oxidation (10). In cultured hepatocytes, CPT-1a protein was down-regulated by the overexpression of miRNA-107 (11). In agreement with above reports, an upregulation of miR107 was found in our models of NAFLD including HFD fed mice and HG treated HepG2 cells. Interestingly, reduction of miR-107 and its target gene, CPT-1a expression level were found in HFD group as well as HepG2 cells treated with HG. These findings along with the fact that miR-107 levels are frequently elevated in several models of NAFLD, suggest that the anti-lipogenic effect of RSV might be partly mediated through the suppression of miR107 in liver cells. It appears that this effect of RSV leads to increased fatty acid β-oxidation, and decreased the availability of fatty acids for triacylglycerol synthesis resulting in lower lipid accumulation in liver cells. According to the bioinformatics analysis, cpt1a is a predicted target gene for miR-107. In cultured hepatocytes, treated with HG, we observed the up regulation miR-107showed the down-regulation of CPT1a gene expression, suggesting that in fact cpt1a is a real target gene for miR-107. In addition, HepG2 cells treated with RSV demonstrated decreased miR-107expression and increased CPT1a gene expression, these results suggest that the increase induced by RSV in CPT1a gene expression, as a molecule involved in fatty acid β-oxidation was mediated by a reduction in miR-107 expression. 

PPARα is a nutritional sensor adapting metabolic homeostasis to energy deprivation (34). It is mostly expressed in the liver, where it regulates lipid catabolism (i.e. β-oxidation) and fatty acid transport (35). Several studies demonstrated that PPARα exerted it influence on hepatic lipid metabolism via activating genes involved in fatty acid oxidation, reducing the hepatic substrate for TG synthesis by limiting its output from other tissues and up regulating the malonyl-COA level for activating the β-oxidation pathway(36, 37). So, the imaginable regulatory axis Twist–miRNA-10b–PPARα–down-stream effector molecule–hepatic lipid metabolism might play an important role in the pathogenesis of NAFLD. Alterations of PPARα expression or activity were associated with various diseases such as obesity and NAFLD (12). It is now clear that deregulations of specific miRNA can significantly contribute to PPARα abnormal signaling in these pathophysiological conditions. In this regards, it was uncovered that miR-10b was upregulated following exposure to fatty acids and had a unique binding site in the PPARα in human hepatic L02 cells (38). To understand the role of miR10b in the anti-lipogenic effect of RSV, we evaluated miR-10b and PPARα expression in both our *In-vivo* and *In-vitro* models. In this study, RSV reduced the expression of miR-10b in HFD-treated mice and HepG2 cells treated with HG, whereas, the expression of PPARα was significantly increased following RSV treatment in both *In-vivo* and *In-vitro* experiments. Taken together, these findings imply that miR10b and its target gene, PPARα could be considered as one of the target molecules by which RSV reduces lipid accumulation in liver cells. 

In summary, our study suggests that the inhibitory effect of RSV on lipid accumulation might be partly mediated through down regulation of miR-107 and miR-10b in both *In-vivo* and *In-vitro* models of NAFLD. Down regulation of these miRNAs in turn could up regulate the expression of their target genes CPT-1a and PPARα genes leading to increasing β-oxidation and suppressing lipid accumulation in liver cells.

**Table 1.** Synthesized primers used in the study

**Gene´s name**	**Primer sequence**
miR-107	5’-CAGCAGCATTGTACAGGGCTAT-3’
miR-10b	5’-TGTACCCTGTAGAACCGAATTTG-3’
mmu-CPT-1a	F: CTCCGCCTGAGCCATGAAG R: CACCAGTGATGATGCCATTCT
mmu-PPARα	F: TACTGCCGTTTTCACAAGTGC R: AGGTCGTGTTCACAGGTAAGA
hsa-CPT-1a	F: ATCAATCGGACTCTGGAAACGG R: TCAGGGAGTAGCGCATGGT
hsa-PPARα	F: ATGGTGGACACGGAAAGCC R: CGATGGATTGCGAAATCTCTTGG
ß-actin	F: AACGGTGCCAAGGAGGATTT R: ATTCCACCAGTGAGTTGCGT
RNAU6	CGC AAG GAT GAC ACG CAA ATT C

hsa: human (*Homo sapiens*), mmu:* Mus musculus*, CPT-1a: Carnitine palmitoyltransferase I, PPARα: Peroxisome proliferator-activated receptor alpha.
